# The Usefulness of the COVID-GRAM Score in Predicting the Outcomes of Study Population with COVID-19

**DOI:** 10.3390/ijerph191912537

**Published:** 2022-10-01

**Authors:** Agata Sebastian, Marcin Madziarski, Marta Madej, Krzysztof Proc, Małgorzata Szymala-Pędzik, Joanna Żórawska, Michał Gronek, Ewa Morgiel, Krzysztof Kujawa, Marek Skarupski, Małgorzata Trocha, Piotr Rola, Jakub Gawryś, Krzysztof Letachowicz, Adrian Doroszko, Barbara Adamik, Krzysztof Kaliszewski, Katarzyna Kiliś-Pstrusińska, Agnieszka Matera-Witkiewicz, Michał Pomorski, Marcin Protasiewicz, Janusz Sokołowski, Ewa Anita Jankowska, Katarzyna Madziarska

**Affiliations:** 1 Department of Rheumatology and Internal Medicine, Wroclaw Medical University, Borowska Street 213, 50-556 Wroclaw, Poland; 2 Department of Rheumatology and Internal Medicine, University Hospital, Borowska Street 213, 50-556 Wroclaw, Poland; 3 Clinical Department of Geriatrics, Wroclaw Medical University, Pasteura 4 Street, 50-367 Wroclaw, Poland; 4 Clinical Department of Angiology, Hypertension and Diabetology, University Hospital, Borowska Street 213, 50-556 Wroclaw, Poland; 5 Statistical Analysis Centre, Wroclaw Medical University, K. Marcinkowski Street 2-6, 50-368 Wroclaw, Poland; 6 Faculty of Pure and Applied Mathematics, Wroclaw University of Science and Technology, Wybrzeze Wyspianskiego Street 27, 50-370 Wroclaw, Poland; 7 Department of Pharmacology, Wroclaw Medical University, Mikulicz-Radecki Street 2, 50-345 Wroclaw, Poland; 8 Department of Cardiology, Provincial Specialized Hospital, Iwaszkiewicza 5 Street, 59-220 Legnica, Poland; 9 Clinical Department of Internal and Occupational Diseases, Hypertension and Clinical Oncology, Wroclaw Medical University, Borowska 213, 50-556 Wroclaw, Poland; 10 Clinical Department of Nephrology and Transplantation Medicine, Wroclaw Medical University, Borowska Street 213, 50-556 Wroclaw, Poland; 11 Clinical Department of Anaesthesiology and Intensive Therapy, Wroclaw Medical University, Borowska Street 213, 50-556 Wroclaw, Poland; 12 Clinical Department of General, Minimally Invasive and Endocrine Surgery, Wroclaw Medical University, Borowska Street 213, 50-556 Wroclaw, Poland; 13 Clinical Department of Paediatric Nephrology, Wroclaw Medical University, Borowska Street 213, 50-556 Wroclaw, Poland; 14 Screening of Biological Activity Assays and Collection of Biological Material Laboratory, Wroclaw Medical University Biobank, Wroclaw Medical University, Borowska Street 211A, 50-556 Wroclaw, Poland; 15 Clinical Department of Gynecology and Obstetrics, Wroclaw Medical University, Borowska Street 213, 50-556 Wroclaw, Poland; 16 Clinical Department and Clinic of Cardiology, Wroclaw Medical University, Borowska Street 213, 50-556 Wroclaw, Poland; 17 Department of Emergency Medicine, Wroclaw Medical University, Borowska Street 213, 50-556 Wroclaw, Poland; 18 Institute of Heart Diseases, Wroclaw Medical University, Borowska Street 213, 50-556 Wroclaw, Poland; 19 Institute of Heart Diseases, University Hospital in Wroclaw, Borowska Street 213, 50-556 Wroclaw, Poland

**Keywords:** GRAM score, COVID-19, mortality

## Abstract

Background: The COVID-GRAM is a clinical risk rating score for predicting the prognosis of hospitalized COVID-19 infected patients. Aim: Our study aimed to evaluate the use of the COVID-GRAM score in patients with COVID-19 based on the data from the COronavirus in the LOwer Silesia (COLOS) registry. Material and methods: The study group (834 patients of Caucasian patients) was retrospectively divided into three arms according to the risk achieved on the COVID-GRAM score calculated at the time of hospital admission (between February 2020 and July 2021): low, medium, and high risk. The Omnibus chi-square test, Fisher test, and Welch ANOVA were used in the statistical analysis. Post-hoc analysis for continuous variables was performed using Tukey’s correction with the Games–Howell test. Additionally, the ROC analysis was performed over time using inverse probability of censorship (IPCW) estimation. The GRAM-COVID score was estimated from the time-dependent area under the curve (AUC). Results: Most patients (65%) had a low risk of complications on the COVID-GRAM scale. There were 113 patients in the high-risk group (13%). In the medium- and high-risk groups, comorbidities occurred statistically significantly more often, e.g., hypertension, diabetes, atrial fibrillation and flutter, heart failure, valvular disease, chronic kidney disease, and obstructive pulmonary disease (COPD), compared to low-risk tier subjects. These individuals were also patients with a higher incidence of neurological and cardiac complications in the past. Low saturation of oxygen values on admission, changes in C-reactive protein, leukocytosis, hyperglycemia, and procalcitonin level were associated with an increased risk of death during hospitalization. The troponin level was an independent mortality factor. A change from low to medium category reduced the overall survival probability by more than 8 times and from low to high by 25 times. The factor with the strongest impact on survival was the absence of other diseases. The medium-risk patient group was more likely to require dialysis during hospitalization. The need for antibiotics was more significant in the high-risk group on the GRAM score. Conclusion: The COVID-GRAM score corresponds well with total mortality. The factor with the strongest impact on survival was the absence of other diseases. The worst prognosis was for patients who were unconscious during admission. Patients with higher COVID-GRAM score were significantly less likely to return to full health during follow-up. There is a continuing need to develop reliable, easy-to-adopt tools for stratifying the course of SARS-CoV-2 infection.

## 1. Introduction

A new coronavirus, SARS CoV2, has widely spread throughout the world since late 2019, causing the COVID-19 pandemic. Worldwide, as of 11 September 2022, 605 million confirmed cases and 6.4 million deaths have been reported. In the first week of September 2022, over 3.1 million new cases were identified [1]. Among the clinical presentation, the virus causes diffuse interstitial pneumonia. Acute hypoxemic respiratory failure is the most common form of organ failure, contributing to over 90% of COVID-19-related deaths among anesthesiology and intensive care patients [2,3]. About a quarter of patients with critical COVID-19 require non-invasive ventilation (NIV), two-thirds require invasive mechanical ventilation (MV), two-thirds require vasopressor support, and one-sixth requires renal replacement therapy [2,4].

In a multivariable Cox regression model, significant risk factors of severe COVID-19 (as defined by hospitalization or death due to COVID-19) were older age (≥65 years vs. 20 to 44 years), men (vs women), Hispanic ethnicity (vs non-Hispanic ethnicity), Black or Asian race (vs White race), obesity (vs normal weight), and an increasing number of chronic conditions. The authors found that the associations of chronic conditions with the risk of severe COVID-19 were stronger for younger adults than for older adults. Among younger adults, particularly strong associations were observed for developmental disorders, personality disorders, affective disorders, schizophrenia, and other psychoses [5]. The other risk factor are malignancies [6]. According to Zhou, some abnormalities in laboratory tests, including decreased lymphocytes and increased serum lactate dehydrogenase (LDH) levels, should also be considered as a marker of poor prognosis [7].

An early stratification of patients with a poor prognosis is extremely important, since it allows for the earlier and targeted application of appropriate therapies. One of the tools proposed to date in stratifying the risk of a severe COVID-19 course is the COVID-GRAM score [8], which was introduced in 2020 to assess more precisely the patients admitted to the hospital. The COVID-GRAM score includes ten independent predictive factors, i.e., pathological changes typical of COVID-19 in chest radiographs, patients’ age, hemoptysis, dyspnea, loss of consciousness, number of comorbidities, history of malignancy, neutrophil-to-lymphocyte ratio, LDH value, and bilirubin concentration. The mortality is the critical outcome for testing the efficacy of therapeutics in hospitalized patients with COVID-19.

In this study, using the data from the COronavirus in LOwer Silesia (COLOS) registry, we analyzed the cohort of polish patients with COVID-19, aiming to assess the diagnostic performance of the COVID-GRAM score for fatal and non-fatal clinical outcomes.

## 2. Materials and Methods

### 2.1. Analyzed Population

**Figure 1:**
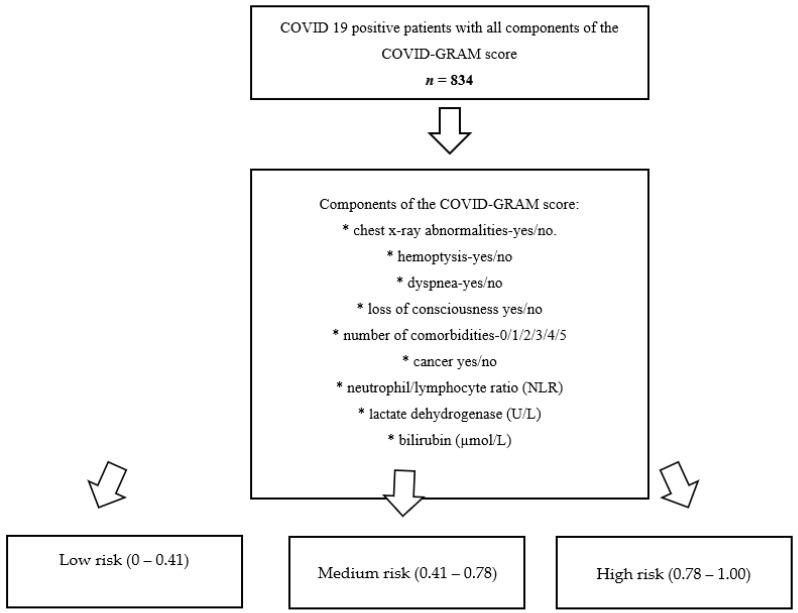
A flowchart of the study protocol presenting the subjects’ recruitment.

The study group consisted of 834 unvaccinated patients with confirmed COVID-19 infection (nasopharyngeal swab—PCR) who, due to infection, required hospitalization between February 2020 and July 2021. The hospital admission criteria included: age ≥ 18 years, the need for oxygen therapy or pharmacological therapy that cannot be used on an outpatient basis (glucocorticosteroids, intravenous antibiotics, baricitinib, remdesivir, blood transfusions). We did not analyze patients who were intubated before hospitalization and needed treatment in Intensive Care Unit (ICU) from the beginning. All clinical data are retrospective and come from the COLOS registry. All patients had the parameters necessary to calculate the GRAM score. The complete study design is shown in detail in Figure 1.

The Bioethics Committee of the Wroclaw Medical University, Poland approved the study (KB number: 444/2021). Written consent for the study was not required from patients due to the retrospective and observational nature of the study.

### 2.2. Clinical Follow-Up and Outcomes

On admission to the hospital, all patients underwent a complete physical examination with saturation assessment and a panel of basic laboratory tests (blood counts, kidney function, liver function, inflammatory parameters) and imaging tests (computed tomography (CT) or chest CT angio) were performed, which were later repeated during the hospitalization depending on the clinical assessment of the patient. The mortality analysis was performed 3 and 6 months after the patients had been discharged from the hospital to establish the primary points.

Secondary outcomes included: the need for mechanical ventilation support, myocardial injury, shock, acute heart failure, pulmonary embolism, stroke, acute kidney injury, acute liver dysfunction, pneumonia, sepsis, systemic inflammatory response syndrome (SIRS), multiple organs dysfunction syndrome (MODS), and bleedings.

### 2.3. Study Groups

The study group was divided into three arms according to the risk achieved on the COVID-GRAM score calculated at the time of hospital admission:

- Low risk: 0–0.41;
- Medium risk: 0.41–0.78;
- High risk: 0.78–1.00.

In further analyses, the designated division was applied.

### 2.4. Statistical Analysis

Descriptive data are presented as numbers and percentages for categorical variables, as mean with standard deviation range (minimum–maximum) and a number of non-missing values for numerical variables for parametrical variables, or as median with interquartile range for non-parametrical variables, respectively. An Omnibus chi-square test was used for categorical variables with more than five expected cases in each group, whereas the Fisher exact test was used for cases with fewer cell counts. Welch’s ANOVA was performed for continuous variables due to unequal variances between the risk strata and sample size large enough for appropriateness of asymptotic results. Post-hoc analysis for continuous variables was performed using the Games–Howell test with a Tukey correction. For categorical variables, the post-hoc test was the same as the Omnibus test. The data on in-hospital and all-cause mortality was available as the right-censored data; thus, time-dependent ROC analysis with Inverse Probability of Censoring Weighting (IPCW) estimation was performed for all those variables. The GRAM-COVID score was assessed through the time-dependent area under the curve (AUC). The area under the ROC curve (AUC) results were considered excellent for AUC values between 0.9–1, good for AUC values between 0.8–0.9, fair for AUC values between 0.7–0.8, poor for AUC values between 0.6–0.7, and failed for AUC values between 0.5–0.6

Log-rank test was used to confirm differences in survival curves between risk strata.

All statistical analyses were performed using the R version 4.0.4 using package time–ROC, pROC [9], survival [10], coin [11], and final fit. A significance level of 0.05 was selected for all statistical analyses.

The calculations were made in R and some of the graphs were also made in MS Excel.

## 3. Results

### 3.1. Patients Baseline Characteristics

**Figure 2:**
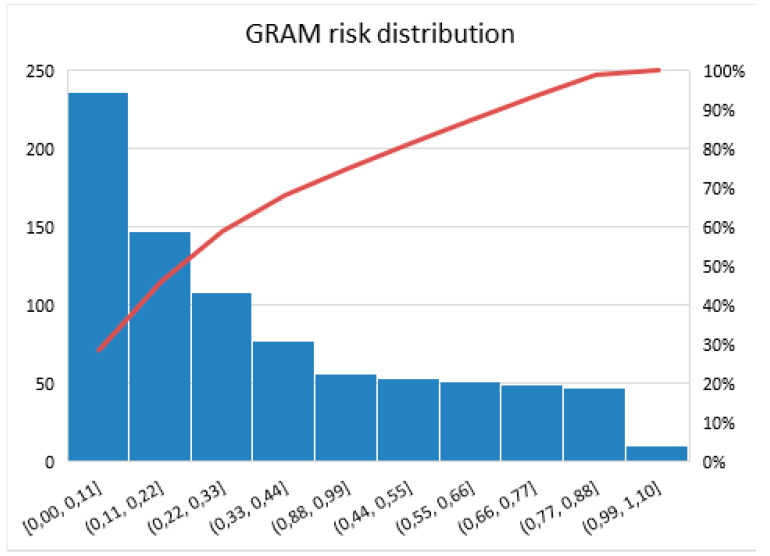
Pareto chart shows the percentage distribution of patients according to risk stratification on the GRAM score in patients with COVID-19 infection. The red line indicates the cumulative number (in percent) of patients with GRAM scores in each range.

Most patients were characterized by a low risk of complications on the COVID-GRAM score (541 patients). The percentage distribution of patients according to risk stratification is shown in Figure 2.

There was no difference in the increased risk of severe disease and endpoints in men, obese subjects, and cigarette smokers. Those in the medium- and the higher-risk group were statistically significantly more likely to have hypertension, diabetes, atrial fibrillation and flutter, heart failure, valvular defects, chronic kidney disease, and obstructive pulmonary disease (COPD) compared to the subjects from the low-risk stratum. These subjects were also patients who were more likely to have a cardiac history of myocardial infarction in the past requiring revascularization procedures. Peripheral artery disease, stroke or transient ischemic attack (TIA) were more common in the high-risk than in the low-risk stratum. The medium-risk patient stratum was more prone to require dialysis during hospitalization than the low-risk patient. The analyzed subgroups did not differ in the incidence of lipid disorders, asthma, and thyroid disease.

Regarding therapies used before hospitalization, the low-risk subgroup was less likely to be on angiotensin-converting enzyme inhibitors (ACEIs), beta-blockers, loop diuretics, statins, novel oral anticoagulants (NOACs), insulin, and proton pump inhibitors. The treatment of chronic diseases did not change during hospitalization and COVID-19 treatment.

Table 1 and Table 2 outline the complete characteristics of the subgroups with respect to the presence of comorbidities and the treatment applied before hospitalization.

During hospital admission, the medium- and high-risk groups were more likely to have lower diastolic blood pressure values and baseline lower saturation (<90%). Crackles over the lung fields on physical examination were more commonly found in the medium-risk group (in 30% of patients). On the other hand, wheezing was more frequently observed in the high-risk group. Congestion over the lungs and peripheral edema were least common in the low-risk group. There was no correlation between the number of COVID-GRAM score and other clinical signs on admission, including body temperature, systolic blood pressure, taste and smell disturbances, and clinical signs of gastrointestinal involvement, among others (Table 3).

### 3.2. Laboratory Test Results

Table 4 shows the results of laboratory tests determined on admission and at the end of hospitalization. Both on admission and at the end of hospitalization, the group of patients with a higher risk showed elevated leukocyte values, lower hemoglobin and platelet values, as well as reduced Estimated Glomerular Filtration Rate (eGFR) values. In patients with the worst prognosis during admission, higher values of C-reactive protein (CRP), procalcitonin, D-dimer, LDH, international normalized ratio (INR), prothrombin, potassium, urea, creatinine, and reduced values of total protein and albumin were found. Higher glucose levels on admission were also observed in this group of patients. Higher exponents of myocardial damage, expressed by B-type natriuretic peptide test (BNP) and N-terminal (NT)-pro hormone BNP (NT-proBNP) parameters and troponin levels, were observed in the high-risk group according to the COVID-GRAM score, both on admission and at the end of hospitalization. The analyzed subpopulations of patients did not differ considering the results of aminotransferases, lipidogram, thyroid-stimulating hormone (TSH), and vitamin D3 levels.

In arterial blood gasometry determined at admission, no significant differences were noted between low-, medium-, and high-risk groups, except for an increased percentage of patients with hypercapnia in the low-risk group relative. This finding seems to be particularly interesting, due to the fact that neither asthma nor COPD occurred more frequently among subjects from the low-risk group.

### 3.3. Therapy Used during Hospitalization

There were no significant differences in all three groups, except for antibiotic therapy, which was used more frequently in the medium and high-risk groups than in the low-risk group. Details of the treatment administered to patients during hospitalization are shown in Table 5.

### 3.4. Clinical Outcome

#### 3.4.1. Correlation between COVID-GRAM Score and Mortality

**Figure 3:**
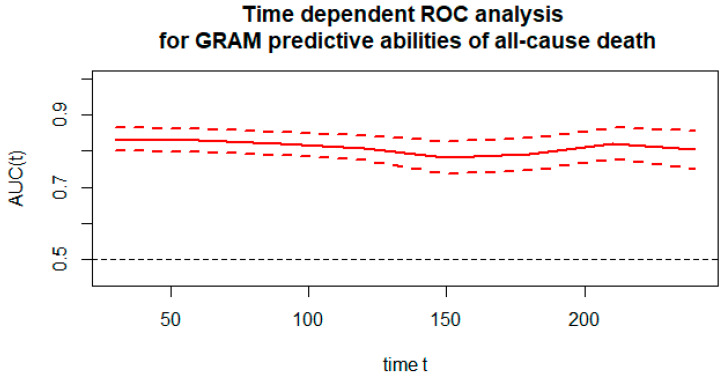
Plot of predictive abilities expressed as area under the ROC curve versus time (in days), along with confidence intervals for this area.

A timeROC analysis was conducted to assess the predictive ability of the COVID-GRAM score of deaths at the time from hospital admission. All causes of death were considered in the analysis, not only due to COVID-19 infection. Figure 3 shows the predictive abilities expressed as the area under the ROC curve versus time and the confidence intervals for this area.

**Figure 4:**
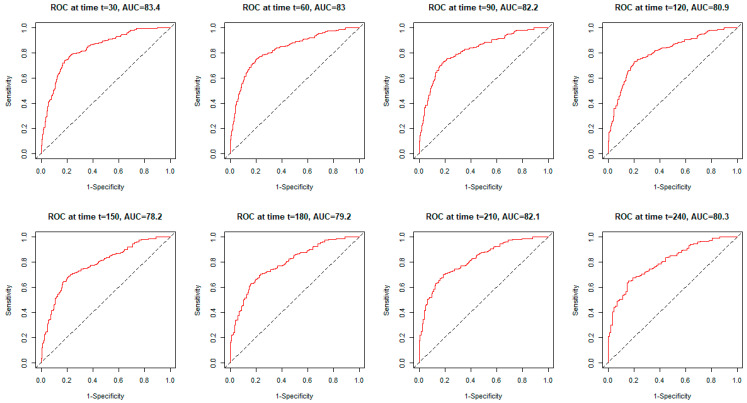
The ROC curves for the GRAM-COVID score for 30–240 days among COVID-19 patients.

During the period studied, the quality of the classification was good. It dropped slightly to the fair class (Fair) in the short term. The AUC level then dropped to 78.2 (Figure 4). Figure 4 shows the ROC curves for the COVID-GRAM score for about 1 to 8 months ahead (30–240 days). The AUC value for the ROC curve at the corresponding time is presented in Figure 4.

**Figure 5:**
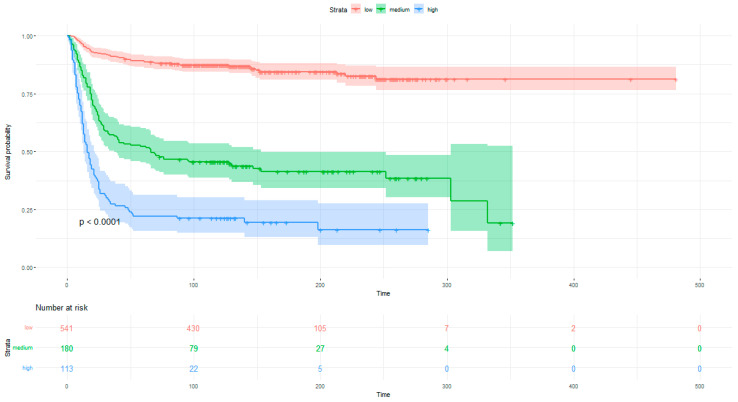
In-hospital survival probability analysis for low-, medium- and high-risk patients with COVID-19 using the COVID-GRAM score.

The survival curves for all COVID-GRAM score levels were determined based on the Kaplan–Meier function. The curves were compared using the log-rank test. The probability of survival is significantly different in the individual risk groups (Figure 5).

Considering the categorized model, a change from the low to medium category reduced the overall survival probability by more than 8 times, whereas from the low to the high stratum by 25 times (Table 6).

The factor with the strongest impact on survival was the absence of other diseases—patients without additional chronic diseases had an 11 times higher chance of survival. The worst prognosis was for patients who were unconscious during admission and whose chance of survival appeared to be 5–6 times lower (Table 7).

The rate of in-hospital death in patients in the medium-risk group was 9.4 times higher than in the low-risk group, and in the high-risk group, it increased as much as more than by 32 times (Table 8).

#### 3.4.2. Correlation between COVID-GRAM Score and the Secondary Endpoints

Correlation between the COVID-GRAM score and the secondary endpoints.

All non-fatal events are shown in Table 9. The all-cause shock was 6 times more common in the medium-risk group and 11 times more common in the high-risk vs. low-risk group, respectively. However, there was no higher incidence of significant deterioration between patients assigned to the different groups. Patients with higher scores were significantly less likely to return to full health during follow-up (4.5×/25×), and were characterized by more frequent decompensations of heart failure (12.7×/27×), neurological disorders (2.4×/3×), pneumonia (4.9×/8.2×), stroke (3.9×/4.9×), and embolism (1.9×/2.4×).

The increase in GRAM scores also increased the incidence of all bleedings (2.9× and 6.3× for the medium- and high-risk groups, respectively). There was no higher incidence of systemic inflammatory response syndrome (SIRS).

## 4. Discussion

In the COVID-19-presenting population, more than 60% of patients had a low risk of complications on the COVID-GRAM score (65%, 541 patients) and 13% were in the high-risk group (113 patients).

There was no difference in the increased risk of severe disease and endpoints in men, obese subjects, and cigarette smokers. Those in the medium- and the high-risk group were statistically significantly more likely to have cardiac, pulmonary (COPD), endocrynological (diabetes), and nephrological (chronic kidney disease) comorbidities compared to the subjects from the low-risk stratum, and needed specific therapies before hospitalization. The medium-risk patient stratum was more prone to require dialysis during hospitalization than the low-risk patient stratum.

During hospital admission, the medium- and high-risk groups were more likely to have lower diastolic blood pressure values and baseline lower oxygen saturation (<90%). In patients with the worst prognosis during admission, higher values of CRP, procalcitonin, D-dimer, LDH, INR, prothrombin, potassium, urea, creatinine, reduced values of total protein and albumin, and a higher glucose level was observed. Higher exponents of myocardial damage were found in the high-risk group according to the COVID-GRAM score, both on admission and at the end of hospitalization. Antibiotic therapy was used more frequently in other groups than the low-risk group.

Considering the categorized model, a change from the low to medium category reduced the overall survival probability by more than 8 times, whereas from the low to the high stratum by 25 times. The factor with the strongest impact on survival was the absence of other diseases—patients without additional chronic diseases had an 11 times higher chance of survival. The worst prognosis was for patients who were unconscious during admission and whose chance of survival appeared to be 5–6 times lower. Patients with higher scores were significantly less likely to return to full health during follow-up, and were characterized by more frequent decompensations of heart failure, neurological disorders, pneumonia, stroke, bleedings, and embolism.

The COVID-GRAM composite scoring system is a clinical risk rating scale for predicting the prognosis of COVID-19-infected hospitalized patients. To date, more than twenty COVID-19 scores have been developed for patient assessment [11,12]. The first results of such risk stratification were promising during the validation period with certainty based on the AUC reaching 0.88. Nevertheless, the modest sample size to determine the scoring risk and the relatively small sample size for verification constitutes important limitations of this tool [8,11]. In a retrospective study of COVID-19 patients, the National Early Warning score, COVID-GRAM, and ISARIC-4C compared index scores (qCSI) in terms of in-hospital mortality, but the advantage of the results relative to each other was undefined [13]. CURB-65 score is one of the most common indicators around the world with the AUC 0.846, compare to COVID-GRAM AUC 0.7 [11]. However, compared with the cited study, the group of patients we analyzed was greater by over 200, and the AUC for the COVID-GRAM score we achieved was 0.8 after 30 days. Hence, in a situation where we do not have all the parameters needed to calculate the CURB-65 alternative, we can use the COVID-GRAM scale.

The COVID-GRAM score is a simple to use method to stratify the course of COVID-19 severity in hospitalized patients. This study presents the GRAM risk stratification in the cohort of patients with COVID-19. The COVID-GRAM score corresponds well with total mortality. Therefore, it may be useful in the risk assessment particularly at the beginning of hospitalization, as the AUC values ranged from 80–83 and fallen down to 78 for days 150 and 180 following the COVID-19 onset.

We have confirmed that the COVID-GRAM score is an independent predictor of the severe course of COVID-19 infection, as was published by other authors in recent months [14,15,16]. Among adult patients in Spain, the COVID-GRAM score was an independent predictor of critical illness with an AUC of 0.779, similar to our analysis and study from Pakistan (AUC 0.8) [15]. In our study, in contrast to cited researchers, we observed a lower percentage of patients with a high risk of COVID-GRAM (13% vs. 23%) [14].

Strong evidence now shows increased risks for people with various health conditions, including chronic kidney disease, diabetes, lung and liver diseases, cardiovascular disease, obesity, immunodeficiency, certain disabilities, and mental health conditions [17]. As in other publications [14,18], a factor in the more severe course of COVID-19 in our study was the coexistence of chronic diseases, especially hypertension, COPD, and chronic kidney disease. These diseases were statistically more frequent in the medium- and high-risk groups in the COVID-GRAM stratification than in the low-risk group; in the high-risk group, they were found in 84% of patients (hypertension), 7.9% (COPD), and 28% (chronic kidney disease). We did not find any mental illnesses in our analyzed population, so we could not determine their impact on the course of COVID-19. This is because hospitalization of patients with mental disorders occurs in specially dedicated departments outside our hospital. However, this impact should be remember, given publications that have demonstrated the critical role of mental disorders in the context of COVID-19 [19]. Early detection and intervention for neurological and mental disorders are urgently needed to control morbidity and mortality from the COVID-19 pandemic. Interestingly, younger patients with mental disorders were associated with higher mortality than elders. For type-specific mental diseases, susceptibility to contracting COVID-19 was associated with pre-existing mood disorders, anxiety, and attention-deficit hyperactivity disorder (ADHD); illness severity was associated with both pre-existing and subsequent mood disorders as well as sleep disturbance, and mortality was associated with pre-existing schizophrenia [19].

Although the exact mechanisms by which pre-existing conditions influence disease susceptibility and severity are not known, inflammatory and hormonal pathways are postulated [20], as well as social factors such as living in crowded or institutionalized settings [19]. It is estimated that one in five people worldwide is at a higher risk of unfavorable COVID-19 outcomes based on chronic disease prevalence [21], demonstrating the importance of creating and validating scales to stratify patients, such as presented in this publication COVID-GRAM. 

While risks generally increase with age and are higher among men [17], we expected to achieve similar results in our population analysis. In our group of COVID-19 patients, we did not confirm the influence of gender on the course of infection, unlike what had been observed in another study [14]. Additionally, increased body temperature was not related to a worse long-term prognosis, unlike in another study [14].

Since the SARS CoV-2 virus mainly affects the respiratory system in hospitalized patients, manifesting itself as lung involvement, in clinical practice, apart from imaging studies, basic ventilation parameters (respiratory rate, saturation, PaO_2_/FiO_2_) are used to assess the patient’s condition. In our study population, we did not find significant differences in the blood gases collected at admission to the hospital between the low-, medium- and high-risk groups on the COVID-GRAM score. One possible explanation for this is that patients classified to medium- and, especially, high-risk groups had been subjected to oxygen therapy previously (had been hyperventilating due to higher degree of dyspnea). We only identified an increased proportion of patients with hypercapnia in the low-risk group versus the medium-risk group. On the other hand, we observed lower oxygen saturation at baseline in the group with medium and high risk according to the COVID-GRAM score. Similarly, in the study by Olivas-Martínez et al., the low values of saturation on admission were associated with an increased risk of death during hospitalization [22]. It is interesting that COPD was a factor of poor prognosis in the medium- and high-risk group according to the score. However, this was not the case for asthma. Our results are consistent with the observations recently reported by Song et al. It is suspected that patients with asthma and COPD are likely to have a different risk of severe COVID-19, which may be related to different angiotensin-converting enzyme II (ACE2) expression [23].

Inflammatory parameters have been used successfully on admission to determine the severity of infection in various diseases. The most commonly determined parameters include CRP, leukocytosis, and procalcitonin. Similarly, in the COVID-19 pandemic, their positive predictive value in the severity of the course of infection was assessed [24,25], which we confirmed in our study. In patients with the worst prognosis during admission, higher values of CRP, procalcitonin, D-dimer, LDH, INR, prothrombin, potassium, urea, creatinine, glucose, and reduced values of total protein and albumin were found. Studies addressing the clinical usefulness of CRP have mostly reported a positive association between disease severity and baseline values. Some authors showed that the CRP level could predict disease worsening among non-severe cases, reporting a 5% risk of developing a severe course for every unit increase in the CRP level [26]. Lu et al. identified independent predictors of death based on a logistic regression model and then compared the predictors by ROC curve analysis. CRP emerged as the best predictor, better than neutrophil count, D-dimer, and platelet count. Additionally, CRP levels in patients who died from COVID-19 were 10-fold higher than those in survivors [27]. Among our patients, procalcitonin was another predictor of a bad prognosis on admission. The PCT level reportedly is increased in patients with severe disease compared with non-severe COVID-19 patients, reflecting bacterial super-infection. PCT levels do not rise above the normal range in patients with non-complicated COVID-19, thereby representing a candidate marker for serious disease progression [28]. D-dimer and PT levels have been assessed in COVID-19 patients to establish their ability to predict a worse outcome too, defined as ARDS development, ICU admission, and death [29]. However, another academic research paper [30] did not confirm the association of PT with disease severity, reporting no differences in the levels of PT, aPTT, and PT-international normalized ratio (INR) among mild disease, severe disease, and control groups. Therefore, it did not surprise us that we recorded more abnormal clotting parameters in the medium and higher risk groups in the medium and high group risk in COVID-GRAM. This indicates the need for further observations in subpopulations of different patients. As in the publication of Cheng et al. [31], we found that baseline kidney markers were independent risk factors for in-hospital death. 

In the presented analysis, we also noticed that in the group of high-risk patients according to the COVID-GRAM score, higher markers of myocardial damage were noted, expressed by the parameters of BNP and NT-proBNP and the concentration of troponin, both at admission and the end of hospitalization. These observations are consistent with other studies in which troponin was an independent mortality factor [32,33,34].

In the meta-analysis published by Rajpal et al., the authors showed that the parameters at admission that indicate a worse prognosis also include LDH and hypoalbuminemia [35], which we also observed in our subpopulations of the analyzed patients. However, we found no impact of the increase in AST on the worse prognosis. We also did not observe any correlation in the GRAM-COVID assessment of study cohort with regard to the baseline vitamin D concentration, although we hypothetically expected such a relationship [36,37].

As we have assumed based on the current publications [35,38,39], patients with chronic diseases had a worse prognosis, especially in relation to cardiovascular diseases, diabetes, chronic kidney disease, and COPD. The medium-risk COVID-GRAM group also required dialysis. Interestingly, risk stratification was not influenced by a history of lipid disorders. It is possible that this is related to the drugs commonly used in this group of patients to lower cholesterol and triglycerides. On the other hand, in the group of patients with the worst long-term prognosis according to the GRAM-COVID score, higher glucose levels were recorded on admission, which was probably related to the severity of the infection and the body’s anti-inflammatory response, and the fact that diabetes itself is a factor in the more severe course of COVID-19. Taking into account chronic diseases and their impact on risk stratification on the COVID-GRAM score, it is not surprising that the group of patients with the best prognosis used ACEI, beta-blockers, loop diuretics, statins, NOAC, insulin, and proton pump inhibitors less before hospitalization.

According to the available literature, bacterial infections as a coinfection with COVID-19 are associated with higher morbidity and mortality. They occur in approximately 9–15% of the SARS-CoV-2 population [18,40]. In the group of patients we analyzed, we found that the need for antibiotics was greater in the high-risk groups according to the COVID-GRAM score than in the low-risk group. As a percentage, in all populations, more than half of the patients required antibacterial treatment, including over 90% of patients in the medium- and high-risk groups according to COVID-GRAM.

Additionally, we analyzed the effect of GRAM score stratification on mortality in the COVID-19 group. Taking the categorized model into account, the change from low to medium reduced overall survival more than 8 times and from low to high 25 times.

Interestingly, the factor that had the strongest impact on survival was the lack of other diseases—patients with one additional chronic disease had as much as 11 times greater chance of survival than other patients. It is also not surprising that patients who were unconscious during admission had the worst prognosis relatively, and their chance of survival turned out to be 5–6 times lower.

Our work was limited by the retrospective nature of the study and the small number of patients in the high-risk COVID-GRAM group. In addition, data were limited to patients from only one study site, which may limit their use in other populations. Apart from that, most of the patients were hospitalized before the introduction of vaccinations in Poland.

## 5. Conclusions

In conclusion, the factor with the strongest impact on survival was the absence of other diseases. The worst prognosis was for patients who were unconscious during admission. Patients with higher COVID-GRAM score were significantly less likely to return to full health during follow-up. The COVID-GRAM score corresponds well with total mortality. There is a continuing need to develop reliable, easy-to-adopt tools for stratifying the course of SARS-CoV-2 infection. As presented in the article, one of such tools can be COVID-GRAM.

## Figures and Tables

**Table 1:** Baseline characteristics of the study cohort after GRAM risk stratification.

Variables, Units (N)	Low Risk	Medium Risk	High Risk	OMNIBUS *p*-Value	*p*-Value for Post-Hoc Analysis
	Mean ± SD Min-Max (N) or n/N (% of Risk Category)	Mean ± SD Min-Max (N) or n/N (% of Risk Category)	Mean ± SD Min-Max (N) or n/N (% of Risk Category)		
**Demographics**					
**Age, years** **(834)**	62.47 ± 15.2 20–97 (541)	72.86 ± 11.8 27–98 (180)	73.14 ± 12.47 23–93 (113)	<0.0001	<0.0001 ^a,b^ 0.979 ^c^
**Age ≥ 65 years** **(834)**	290/541 (53.6%)	146/180 (81.11%)	94/113 (83.19%)	<0.0001	<0.0001 ^a,b^ 1.0 ^c^
**Male gender** **(834)**	305/541 (56.38%)	96/180 (53.33%)	66/113 (58.41%)	0.6648	N/A
**BMI, kg/m^2^** **(270)**	28.6 ± 5.45 19.1–47.75 (159)	29.00 ± 5.36 17.28–45.82 (69)	29.74 ± 5.55 20.02–48.21 (42)	0.4852	N/A
**Obesity** **(BMI ≥ 30 kg/m^2^)** **(270)**	58/159 (36.48%)	31/69 (44.93%)	21/42 (50.0%)	0.2846	N/A
**Cigarette smoking** **never/previous/current** **(834)**	470/541 (87.2%) 44/541 (8.16%) 25/541 (4.64%)	159/180 (88.83%) 11/180 (6.15%) 9/180 (5.03%)	103/113 (91.15%) 7/113 (6.19%) 3/113 (2.65%)	0.701	N/A
**Comorbidities**					
**Hypertension** **(834)**	294/541 (54.34%)	135/180 (75.0%)	96/113 (84.96%)	<0.0001	<0.0001 ^a,b^ 0.1787 ^c^
**DM** **(834)**	113/541 (20.89%)	63/180 (35.0%)	53/113 (46.9%)	<0.0001	0.0061 ^a^ <0.0001 ^b^ 0.0559 ^c^
**Dyslipidemia** **(490)**	192/283 (67.84%)	95/119 (79.83%)	72/88 (81.82%)	0.0062	0.0632 ^a^ 0.0502 ^b^ 1.0 ^c^
**AF/AFL** **(834)**	63/541 (11.65%)	39/180 (21.67%)	41/113 (36.28%)	<0.0001	0.0039 ^a^ <0.0001 ^b^ 0.0281 ^c^
**Previous coronary revascularization** **(834)**	30/541 (5.55%)	26/180 (14.44%)	28/113 (24.78%)	<0.0001	0.0006 ^a^ <0.0001 ^b^ 0.1165 ^c^
**Previous MI** **(834)**	37/541 (6.84%)	30/180 (16.67%)	37/113 (32.74%)	<0.0001	0.0005 ^a^ <0.0001 ^b^ 0.007 ^c^
**HF** **(834)**	41/541 (7.58%)	49/180 (27.22%)	45/113 (39.82%)	<0.0001	<0.0001 ^a,b^ 0.1019 ^c^
**Moderate or severe valvular heart disease or previous valve heart surgery** **(834)**	20/541 (3.7%)	18/180 (10.0%)	16/113 (14.16%)	<0.0001	0.0061 ^a^ <0.0001 ^b^ 1.0 ^c^
**PAD** **(834)**	25/541 (4.62%)	8/180 (4.44%)	14/113 (12.39%)	0.0037	1.0 ^a^ 0.0094 ^b^ 0.0671 ^c^
**Previous stroke/TIA** **(834)**	39/541 (7.21%)	22/180 (12.22%)	21/113 (18.58%)	0.0005	0.1575 ^a^ 0.0008 ^b^ 0.5522 ^c^
**CKD** **(834)**	43/541 (7.95%)	40/180 (22.22%)	32/113 (28.32%)	<0.0001	<0.0001 ^a, b^ 0.8944 ^c^
**Hemodialysis** **(834)**	12/541 (2.22%)	14/180 (7.78%)	5/113 (4.42%)	0.0033	0.0052 ^a^ 0.5754 ^b^ 0.9986 ^c^
**Asthma** **(834)**	28/541 (5.18%)	6/180 (3.33%)	4/113 (3.54%)	0.5054	N/A
**COPD** **(834)**	15/541 (2.77%)	14/180 (7.78%)	9/113 (7.96%)	0.0036	0.0183 ^a^ 0.0499 ^b^ 1.0 ^c^
**Thyroid disease, none/hypothyroidism/** **hyperthyroidism,** **(834)**	482/541 (89.09%) 52/541 (9.61%) 7/541 (1.29%)	154/180 (85.56%) 22/180 (12.22%) 4/180 (2.22%)	105/113 (92.92%) 8/113 (7.08%) 0/113 (0%)	0.3153	N/A

Continuous variables are presented as: mean ± SD, range (minimum–maximum), and number of non-missing values. Categorized variables are presented as: a number with a percentage. Information about the numbers with valid values is provided in the left column. Abbreviations: N—valid measurements, n—number of patients with parameter above cut-off point, SD—standard deviation, OMNIBUS—analysis of variance, BMI—body mass index, DM—Diabetes mellitus, AF/AFL—Atrial fibrillation/flutter, MI—myocardial infarction, HF—Heart failure, PAD—Peripheral artery disease, TIA—transient ischemic attack, CKD—Chronic kidney disease, COPD—Chronic obstructive pulmonary disease, N/A—non-applicable, ^a^—low risk vs. medium risk, ^b^—low risk vs. high risk, ^c^—medium risk vs. high risk.

**Table 2:** Baseline characteristics of the study cohort—treatment applied before hospitalization.

Variables. Units (N)	Low Risk	Medium Risk	High Risk	OMNIBUS *p*-Value	*p*-Value for Post-Hoc Analysis
	n/N (% of Risk Category)	n/N (% of Risk Category)	n/N (% of Risk Category)		
**Treatment applied before hospitalization**					
**ACEI** **(834)**	113/541 (20.89%)	62/180 (34.44%)	38/113 (33.63%)	0.0002	0.0011 ^a^ 0.0153 ^b^ 1.0 ^c^
**ARBs** **(834)**	65/541 (12.01%)	10/180 (5.56%)	10/113 (8.85%)	0.0405	0.0614 ^a^ 1.0 ^b,c^
**MRAs** **(834)**	35/541 (6.47%)	19/180 (10.56%)	12/113 (10.62%)	0.1104	N/A
**Sacubitril/valsartan** **(834)**	1/541 (0.18%)	1/180 (0.56%)	1/113 (0.88%)	0.2833	N/A
**β-blocker** **(834)**	171/541 (31.61%)	81/180 (45.0%)	55/113 (48.67%)	0.0001	0.0045 ^a^ 0.0023 ^b^ 1.0 ^c^
**Digitalis glycoside** **(834)**	6/541 (1.11%)	1/180 (0.56%)	1/113 (0.88%)	0.877	N/A
**Calcium channel blocker** **(non-dihydropiridines)** **(834)**	14/541 (2.59%)	6/180 (3.33%)	1/113 (0.88%)	0.4811	N/A
**Calcium channel blocker (dihydropiridines)** **(834)**	100/541 (18.48%)	35/180 (19.44%)	20/113 (17.7%)	0.9277	N/A
**α-adrenergic blocker** **(834)**	37/541 (6.84%)	16/180 (8.89%)	12/113 (10.62%)	0.326	N/A
**Thiazide or thiazide-like diuretic** **(834)**	52/541 (9.61%)	19/180 (10.56%)	11/113 (9.73%)	0.9338	N/A
**Loop diuretic** **(834)**	52/541 (9.61%)	32/180 (17.78%)	24/113 (21.24%)	0.0003	0.0142 ^a^ 0.0025 ^b^ 1.0 ^c^
**Statin** **(834)**	11/541 (20.52%)	60/180 (33.33%)	39/113 (34.51%)	0.0001	0.002 ^a^ 0.0059 ^b^ 1.0 ^c^
**Acetylsalicylic acid** **(834)**	82/541 (15.16%)	35/180 (19.44%)	28/113 (24.78%)	0.0351	0.6508 ^a^ 0.0565 ^b^ 1.0 ^c^
**The second antiplatelet drug—P_2_Y_12_ inhibitor** **(834)**	7/541 (1.29%)	5/180 (2.78%)	5/113 (4.42%)	0.0626	N/A
**LMWH** **(834)**	47/541 (8.69%)	15/180 (8.33%)	16/113 (14.16%)	0.1668	N/A
**VKA** **(834)**	13/541 (2.4%)	7/180 (3.89%)	3/113 (2.65%)	0.5427	N/A
**NOAC** **(834)**	31/541 (5.73%)	11/180 (6.11%)	15/113 (13.27%)	0.014	1.0 ^a^ 0.0241 ^b^ 0.1772 ^c^
**Insulin** **(834)**	31/541 (5.73%)	14/180 (7.78%)	17/113 (15.04%)	0.0027	1.0 ^a^ 0.0034 ^b^ 0.2286 ^c^
**Metformin** **(834)**	72/541 (13.31%)	35/180 (19.44%)	21/113 (18.58%)	0.0834	N/A
**SGLT2 inhibitor** **(834)**	9/541 (1.66%)	2/180 (1.11%)	3/113 (2.65%)	0.558	N/A
**Oral antidiabetics other than SGLT2 inhibitor and metformin** **(834)**	26/541 (4.81%)	12/180 (6.67%)	12/113 (10.62%)	0.0553	N/A
**Proton pump inhibitor** **(834)**	70/541 (12.94%)	45/180 (25.0%)	21/113 (18.58%)	0.0005	0.0006 ^a^ 0.4603 ^b^ 0.7679 ^c^
**Oral corticosteroid** **(834)**	33/541 (6.1%)	6/180 (3.33%)	11/113 (9.73%)	0.079	N/A
**Immunosuppression other than oral corticosteroid** **(834)**	24/541 (4.44%)	9/180 (5.0%)	6/113 (5.31%)	0.8985	N/A

Categorized variables are presented as: a number with a percentage. Information about the numbers with valid values is provided in the left column. Abbreviations: N—valid measurements, N—number of patients with parameter above cut-off point, OMNIBUS—analysis of variance, ACEI—angiotensin-converting-enzyme inhibitors, ARBs—angiotensin receptor blockers, MRAs—mineralocorticoid receptor antagonists LMWH—low molecular weight heparin, VKA—vitamin K antagonists, NOAC—novel oral anticoagulants, SGLT2 inhibitors—sodium glucose cotransporter-2 inhibitors, N/A—non-applicable, ^a^—low risk vs. medium risk, ^b^—low risk vs. high risk, ^c^—medium risk vs. high risk.

**Table 3:** Patient-reported symptoms, vital signs, and abnormalities measured during physical examination at hospital admission in the studied cohort after GRAM risk stratification.

Variables, Units (N)	Low Risk	Medium Risk	High Risk	OMNIBUS *p*-Value	*p*-Value for Post-Hoc Analysis
	Mean ± SD Min-Max (N) or n/N (% of Risk Category)	Mean ± SD Min-Max (N) or n/N (% of Risk Category)	Mean ± SD Min-Max (N) or n/N (% of Risk Category)		
**Patient-reported symptoms**					
**Cough** **(834)**	211/541 (39.0%)	61/180 (33.89%)	35/113 (30.97%)	0.1797	N/A
**Dyspnea** **(834)**	250/541 (46.21%)	96/180 (53.33%)	65/113 (57.52%)	0.043	0.3487 ^a^ 0.1112 ^b^ 1.0 ^c^
**Chest pain** **(834)**	44/541 (8.13%)	6/180 (3.33%)	10/113 (8.85%)	0.0744	N/A
**Hemoptysis** **(834)**	2/541 (0.37%)	2/180 (1.11%)	3/113 (2.65%)	0.0297	0.7825 ^a^ 0.1156 ^b^ 1.0 ^c^
**Smell dysfunction** **(834)**	26/541 (4.81%)	4/180 (2.22%)	2/113 (1.77%)	0.1837	N/A
**Taste dysfunction** **(834)**	23/541 (4.25%)	3/180 (1.67%)	3/113 (2.65%)	0.29	N/A
**Abdominal pain** **(834)**	33/541 (6.1%)	12/180 (6.67%)	4/113 (3.54%)	0.5047	N/A
**Diarrhea** **(834)**	48/541 (8.87%)	11/180 (6.11%)	6/113 (5.31%)	0.2787	N/A
**Nausea and/or vomiting** **(834)**	19/541 (3.51%)	10/180 (5.56%)	4/113 (3.54%)	0.4676	N/A
**Measured vital signs**					
**Body temperature** **°C** **(546)**	37.0 ± 0.9 35.3–40.0 (365)	36.84 ± 0.83 34.4–40.0 (114)	37.16 ± 1.0 35.9–40.5 (67)	0.0611	N/A
**Heart rate** **beats/minute** **(762)**	85.27 ± 14.37 48–139 (493)	83.72 ± 16.98 54–150 (167)	85.35 ± 16.09 50–140 (102)	0.5588	N/A
**Respiratory rate breaths/minute** **(105)**	18.22 ± 4.59 12–30 (46)	20.31 ± 7.23 12–50 (32)	19.26 ± 6.13 12–45 (27)	0.3298	N/A
**SBP** **mmHg** **(772)**	135.62 ± 20.7 80–210 (499)	131.07 ± 22.87 60–200 (170)	133.06 ± 31.82 60–270 (103)	0.0669	N/A
**DBP** **mmHg** **(767)**	79.82 + 12.11 40–143 (498)	75.02 ± 13.34 40–110 (168)	74.37 ± 19.55 40–150 (101)	<0.0001	0.0001 ^a^ 0.022 ^b^ 0.953 ^c^
**SpO2 on room air, % (FiO2 = 21%)** **(500)**	92.01 ± 6.35 48–99 (349)	88.32 ± 10.42 55–99 (100)	88.37 ± 8.37 60–100 (51)	0.0002	0.003 ^a^ 0.011 ^b^ 0.999 ^c^
**Abnormalities detected during physical examination**					
**Crackles** **(834)**	96/541 (17.74%)	59/180 (32.78%)	29/113 (25.66%)	<0.0001	0.0001 ^a^ 0.2083 ^b^ 0.7356 ^c^
**Wheezing** **(834)**	59/541 (10.91%)	30/180 (16.67%)	24/113 (21.24%)	0.0054	0.1705 ^a^ 0.0133 ^b^ 1.0 ^c^
**Pulmonary congestion** **(834)**	121/541 (22.37%)	66/180 (36.67%)	35/113 (30.97%)	0.0005	0.0007 ^a^ 0.2012 ^b^ 1.0 ^c^
**Peripheral edema** **(834)**	49/541 (9.06%)	33/180 (18.33%)	25/113 (22.12%)	<0.0001	0.0033 ^a^ 0.0004 ^b^ 1.0 ^c^

Continuous variables are presented as: mean ± SD, range (minimum–maximum) and number of non-missing values. Categorized variables are presented as: a number with a percentage. Information about the numbers with valid values is provided in the left column. Abbreviations: SD—standard deviation, OMNIBUS—analysis of variance, N—valid measurements, n—number of patients with parameter above cut-off point, SBP—Systolic blood pressure, DBP—Diastolic blood pressure, a—low risk vs. medium risk, b—low risk vs. high risk, c—medium risk vs. high risk.

**Table 4:** Laboratory parameters measured during the hospitalization in the studied cohort.

Parameter (N)	Time of Assessment	Units	Low Risk	Medium	High Risk	OMNIBUS *p*-Value	*p*-Value for Post-Hoc Analysis
			Mean ± SD Min-Max (N) or n/N (% of Risk Category) (N)	Mean ± SD Min-Max (N) or n/N (% of Risk Category) (N)	Mean ± SD Min-Max (N) or n/N (% of Risk Category) (N)		
**Complete Blood Count (CBC)**							
**Leucocytes** **(834)**	On admission	10^3^/µL	7.38 ± 3.78 1.24–35.06 (541)	9.19 ± 5.77 1.16–40.79 (180)	14.69 ± 23.91 0.56–188.7 (113)	<0.0001	0.0003 ^a^ 0.004 ^b^ 0.046 ^c^
	On discharge		8.39 ± 4.77 0.44–53.06 (541)	11.35 ± 7.34 1.19–42.34 (180)	15.27 ± 11.22 1.75–62.67 (113)	<0.0001	<0.0001 ^a,b^ 0.003 ^c^
**Lymphocytes** **(818)**	On admission	10^3^/µL	1.13 ± 0.61 0.09–5.51 (526)	1.0 ± 0.98 0.1–10.95 (180)	1.9 ± 7.71 0.14–78.58 (112)	0.1232	N/A
	On discharge		1.67 ± 0.88 0.1–9.03 (526)	1.32 ± 2.08 0.14–26.71 (180)	1.69 ± 6.39 0.05–66.97 (112)	0.0969	N/A
**Hemoglobin** **(834)**	On admission	g/dL	13.14 ± 2.25 4.3–18.8 (541)	12.35 ± 2.22 3.9–16.8 (180)	12.27 ± 2.17 7.2–17.9 (113)	<0.0001	0.0002 ^a,^ 0.0005 ^b^ 0.943 ^c^
	On discharge		12.61 ± 2.17 7.3–17.9 (541)	11.32 ± 2.23 6.5–17.4 (180)	10.85 ± 2.11 6.0–16.6 (113)	<0.0001	<0.0001 ^a, b^ 0.17 ^c^
**Platelets** **(834)**	On admission	10^3^/µL	233.54 ± 108.64 10–705 (541)	224.16 ± 119.74 5–838 (180)	204.68 ± 96.65 0–537 (113)	0.0188	0.621 ^a^ 0.015 ^b^ 0.279 ^c^
	On discharge		300.27 ± 133.53 13–929 (541)	231.9 ± 116.97 4–592 (180)	193.87 ± 124.99 6–675 (113)	<0.0001	<0.001 ^a,b^ 0.027 ^c^
**Acid-base balance in the arterial blood gas**							
**pH** **(191)**	On admission		7.44 ± 0.05 7.26–7.54 (95)	7.4 ± 0.1 7.04–7.54 (60)	7.4 ± 0.09 7.09–7.58 (36)	0.0033	0.037 ^a^ 0.023 ^b^ 0.927 ^c^
**PaO_2_** **(191)**	On admission	<60 mmHg respiratory insufficiency	23/95 (24.21%)	25/60 (41.67%)	18/36 (50.0%)	0.0081	0.1043 ^a^ 0.0256 ^b^ 1.0 ^c^
			77.9 ± 34.53 27.5–100 (95)	72.83 ± 39.51 33.4–100 (60)	72.07 ± 44.92 23.7–100 (36)	0.6296	N/A
**PaCO_2_** **(191)**	On admission	≥45 mmHg hypercapnia	9/95 (9.47%)	15/60 (25.0%)	5/36 (13.89%)	0.0311	0.0527 ^a^ 1.0 ^b^ 0.8975 ^c^
			35.98 ± 6.36 25.2–56.7 (95)	39.23 ± 13.28 19.7–88.4 (60)	38.21 ± 10.06 25.7–74.9 (36)	0.1338	N/A
**HCO_3_ standard** **(187)**	On admission	mmol/L	24.82 ± 2.68 15.1–32.8 (36)	23.87 ± 4.35 12.1–32.4 (59)	23.16 ± 4.33 15.6–32.9 (92)	0.0573	N/A
**BE** **(65)**	On admission	mmol/L	1.6 ± 3.36 (-)7.7–10.5 (35)	1.38 ± 4.22 (-)7.8–9.7 (18)	0.56 ± 3.17 (-)3.3–6.3 (12)	0.6367	N/A
**Lactates** **(171)**	On admission	mmol/L	2.16 ± 1.01 0.6–6.0 (81)	2.41 ± 1.66 0.5–12.8 (56)	3.11 ± 2.45 0.8–12.0 (34)	0.0792	N/A
**Electrolytes, inflammatory and kidney and liver biomarkers**							
**Na** **(834)**	On admission	mmol/L	137.67 ± 4.66 108–152 (541)	138.64 ± 5.91 113–158 (180)	139.28 ± 8.08 119–175 (113)	0.0265	0.114 ^a^ 0.106 ^b^ 0.743 ^c^
**K** **(834)**	On admission	mmol/L	4.11 ± 0.63 2.6–7.5 (541)	4.32 ± 0.74 2.9–6.9 (180)	4.39 ± 0.77 2.6–6.9 (113)	<0.0001	0.002 ^a^ 0.001 ^b^ 0.723 ^c^
**CRP** **(834)**	On admission	mg/L	74.86 ± 76.55 0.29–428.88 (541)	98.13 ± 84.56 0.42–431.65 (180)	118.22 ± 93.45 0.4–487.38 (113)	<0.0001	0.003 ^a^ <0.0001 ^b^ 0.154 ^c^
**Procalcitonin** **(811)**	On admission	ng/mL	0.53 ± 2.74 0.08–42.19 (522)	3.01 ± 16.59 0.01–196.04 (176)	2.96 ± 10.71 0.02–72.61 (113)	0.0098	0.122 ^a^ 0.048 ^b^ 0.999 ^c^
**IL-6** **(496)**	On admission	pg/mL	43.69 ± 94.85 2–1000 (339)	57.93 ± 116.06 2–1000 (103)	251.42 ± 1231.24 2.94–9099 (54)	0.2548	N/A
**D-dimer** **(775)**	On admission	µg/L	3.11 ± 10.47 0.18–123.93 (500)	6.46 ± 16.63 0.22–128 (166)	10.48 ± 25.09 0.2–132.82 (109)	0.0011	0.04 ^a^ 0.009 ^b^ 0.307 ^c^
**Protrombin rate** **(801)**	On admission	%	82.91 ± 18.13 7–142 (517)	75.04 ± 22.22 5–130 (173)	75.83 ± 21.51 3–128 (111)	<0.0001	0.0001 ^a^ 0.004 ^b^ 0.953 ^c^
**INR** **(801)**	On admission	>1.5	23/517 (4.45%)	23/173 (13.29%)	16/111 (14.41%)	<0.0001	0.0003 ^a^ 0.0004 ^b^ 1.0 ^c^
**aPTT** **(772)**	On admission	>60 s	9/493 (1.83%)	9/169 (5.34%)	4/110 (3.64%)	0.0419	0.0756 ^a^ 0.8087 ^b^ 1.0 ^c^
**Fibrinogen** **(246)**	On admission	g/dL	4.51 ± 1.64 1.23–9.26 (110)	5.02 ± 1.82 0.35–9.2 (73)	4.89 ± 2.05 0.44–9.94 (63)	0.1306	N/A
**Glucose** **(773)**	On admission	mg/dL	133.28 ± 77.19 53.0–933.0 (494)	154.13 ± 99.94 49.0–1064.0 (171)	185.68 ± 11.28 76.0–733 (108)	<0.0001	0.036 ^a^ <0.0001 ^b^ 0.046 ^c^
**Glycated hemoglobin (HbA1c)** **(151))**	On admission	%	7.61 ± 2.2 4.9–16.6 (102)	7.34 ± 1.5 5.4–12.1 (30)	7.31 ± 1.56 5.1–11.7 (19)	0.6536	N/A
**Urea** **(823)**	On admission	mg/dL	46.84 ± 39.58 8.0–336.0 (531)	70.23 ± 50.48 15–298 (179)	80.96 ± 44.13 16.0256.0 (113)	<0.0001	<0.0001 ^a,b^ 0.137 ^c^
**Creatinine** **(834)**	On admission	mg/dL	1.23 ± 1.36 0.38–12.66 (541)	1.63 ± 1.5 0.44–9.49 (180)	1.67 ± 1.07 0.48–7.81 (113)	<0.0001	0.006 ^a^ 0.0006 ^b^ 0.946 ^c^
	On discharge		1.11 ± 1.05 0.44–12.35 (541)	1.43 ± 1.47 0.43–8.48 (180)	1.7 ± 1.34 0.43–9.06 (113)	<0.0001	0.016 ^a^ <0.0001 ^b^ 0.25 ^c^
**eGFR** **(834)**	On admission	**mL/min/** **1.73 m^2^**	**78.45 ± 30.48** **3.0–250.0** (541)	**62.34 ± 33.85** **6.0–183.0** (180)	**53.92 ± 32.71** **5.0–196.0** (113)	<0.0001	<0.0001 ^a,b^ 0.074 ^c^
	On discharge		**84.0 ± 30.31** **4.0–212.0** (541)	**74.44 ± 40.47** **6.0–226.0** (180)	**63.55 ± 42.06** **4.0–209.0** (113)	<0.0001	0.011 ^a^ <0.0001 ^b^ 0.075 ^c^
**Total protein** **(371)**	On admission	g/L	6.1 ± 0.87 3.6–9.5 (225)	5.7 ± 0.85 3.4–7.9 (90)	5.54 ± 0.69 4.2–7.2 (56)	<0.0001	0.0009 ^a^ <0.0001 ^b^ 0.396 ^c^
**Albumin** **(419)**	On admission	g/L	3.24 ± 0.57 1.5–4.9 (220)	2.91 ± 0.53 1.1–4.1 (118)	2.94 ± 0.51 0.7–4.4 (81)	<0.0001	<0.0001 ^a,b^ 0.972 ^c^
**AST** **(825)**	On admission	IU/L	54.31 ± 78.31 6.0–1261 (533)	53.57 ± 47.8 11–378 (180)	133.04 ± 351.16 10.0–2518.0 (112)	0.0611	N/A
**ALT** **(828)**	On admission	IU/L	49.29 ± 85.56 4–1278 (536)	41.12 ± 49.12 5–455.0 (180)	78.67 ± 204.2 5.0–1411.0 (112)	0.0687	N/A
**Bilirubin** **(834)**	On admission	U/L	0.73 ± 0.61 0.1–7.9 (541)	0.77 ± 0.64 0.2–6.6 (180)	0.95 ± 1.19 0.1–10.0 (113)	0.1258	N/A
**LDH** **(834)**	On admission	U/L	361.77 ± 168.55 44–1175 (541)	441.84 ± 233.6 71–1609 (180)	807.64 ± 1153.37 151.0–9505.0 (113)	<0.0001	<0.0001 ^a^ 0.0003 ^b^ 0.003 ^c^
**Cardiac biomarkers**							
**BNP** **(267)**	On admission	pg/mL	354.62 ± 1015.43 1.7–7954.2 (132)	739.65 ± 2104.97 8.0–13368.4 (73)	685.2 ± 899.28 10.5–4993.0 (62)	0.0472	0.31 ^a^ 0.061 ^b^ 0.978 ^c^
	On discharge		340.72 ± 994.11 1.7–7954.2 (132)	674.08 ± 1986.29 8.0–13368.4 (73)	642.12 ± 765.72 10.5–2779.5 (62)	0.0526	N/A
**NT-proBNP** **(200)**	On admission	ng/mL	3484.78 ± 10448.54 18.2–70000 (100)	9452.86 ± 16351.53 29.7–70000 (60)	12746.17 ± 18013.17 63.1–70000 (40)	0.0019	0.035 ^a^ 0.01 ^b^ 0.624 ^c^
	On discharge		4019.58 ± 11325.74 18.2–70000 (100)	8277.73 ± 14383.38 29.7–70000 (60)	12628.59 ± 16894.12 149.1–70000 (40)	0.0067	0.128 ^a^ 0.012 ^b^ 0.379 ^c^
**Troponin T** **normal value:** **F < 15.6 pg/mL** **M < 34.2 pg/mL** **(516)**	On admission	pg/mL	181.91 ± 1351.26 1.0–21022.9 (294)	376.86 ± 1548.68 0.2–14128.8 (130)	1349.49 ± 5550.69 3.2–48854.9 (92)	0.0769	N/A
		>5-fold upper range K 46.8 M 102.6	37/294 (12.59%)	34/130 (26.15%)	44/92 (47.83%)	<0.0001	0.0028 ^a^ <0.0001 ^b^ 0.0043 ^c^
		>3-fold upper range K 46.8 M 102.6	48/294 (16.33%)	53/130 (40.77%)	54/92 (58.7%)	<0.0001	<0.0001 ^a,b^ 0.0376 ^c^
	On discharge	pg/mL	703.62 ± 10197.17 0.8–174652.6 (294)	237.54 ± 1365.37 0.2–15223.1 (130)	1057.89 ± 3013.31 3.2–17408.1 (92)	0.0449	0.723 ^a^ 0.858 ^b^ 0.042 ^c^
**LDL-cholesterol** **(260)**	On admission	mg/dL	87.54 ± 44.29 6–248 (184)	85.02 ± 39.75 23–187 (47)	92.45 ± 51.38 25–215 (29)	0.8011	N/A
**HDL-cholesterol** **(260)**	On admission	mg/dL	37.84 ± 15.28 2–110 (184)	37.09 ± 12.18 8–65.0 (47)	35.76 ± 14.62 16–79 (29)	0.7621	N/A
**TG** **(388)**	On admission	mg/dL	143.44 ± 75.04 44–575 (222)	175.67 ± 123.75 50–637 (93)	185.77 ± 130.27 50–760 (73)	0.0049	0.054 ^a^ 0.027 ^b^ 0.868 ^c^
**Hormones**							
**25-hydroxy-vitamin D** **(345)**	On admission	ng/mL	23.92 ± 15.95 3.5–126.4 (227)	22.54 ± 15.53 3.5–73.2 (70)	18.82 ± 13.49 3.5–75.6 (48)	0.0755	N/A
**TSH** **(432)**	On admission	mIU/L	1.6 ± 2.64 0–28.81 (276)	1.31 ± 1.33 0–8.28 (100)	1.54 ± 2.01 0.03–11.16 (56)	0.3654	N/A

Continuous variables are presented as: mean ± SD, range (minimum–maximum), and number of non-missing values. Categorized variables are presented as: a number with a percentage. Information about the numbers with valid values is provided in the left column. Abbreviations: N—valid measurements, n—number of patients with parameter above cut-off point, OMNIBUS—analysis of variance, SD—standard deviation, N/A—non-applicable, PaO_2_—partial pressure of oxygen, PaCO_2_—partial pressure of carbon dioxide, HCO_3_—bicarbonate ion, BE—base excess, Na—natrium, K—kalium, CRP—C-reactive protein, IL-6—interleukin 6, INR—international normalized ratio, aPTT—activated partial thromboplastin time, eGFR—estimated glomerular filtration rate, AST—aspartate transaminase, ALT—Alanine transaminase, LDH—Lactate dehydrogenase, BNP—B-type natriuretic peptide, NT-pro BNP—N-terminal prohormone of brain natriuretic peptide, LDL—Low-density lipoprotein, HDL—High-density lipoprotein, TG—triglyceride, TSH—Thyroid-stimulating hormone; a—low risk vs. medium risk, b—low risk vs. high risk, c—medium risk vs. high risk.

**Table 5:** Therapies applied during the hospitalization in the studied cohort.

Variables. Units (N)	Low Risk	Medium Risk	High Risk	OMNIBUS *p*-Value	*p*-Value for Post-Hoc Analysis
	n/N (% of Risk Category)	n/N (% of Risk Category)	n/N (% of Risk Category)		
**Applied treatment and procedures**					
**Systemic corticosteroid** **(834))**	385/541 (71.16%)	146/180 (81.11%)	82/113 (72.57%)	0.0314	0.0346 ^a^ 1.0 ^b^ 0.3499 ^c^
**Convalescent plasma** **(834)**	109/541 (20.15%)	38/180 (21.11%)	22/113 (19.47%)	0.9377	N/A
**Tocilizumab** **(834)**	8/541 (1.48%)	5/180 (2.78%)	1/113 (0.88%)	0.4734	N/A
**Remdesivir** **(834)**	164/541 (30.31%)	56/180 (31.11%)	25/113 (22.12%)	0.1868	N/A
**Antibiotic** **(834)**	380/541 (70.24%)	166/180 (92.22%)	105/113 (92.92%)	<0.0001	<0.0001 ^a,b^ 1.0 ^c^

Categorized variables are presented as: a number with a percentage. Information about the numbers with valid values is provided in the left column. Abbreviations: N—valid measurements, n—number of patients with parameter above cut-off point, OMNIBUS—analysis of variance, SD—standard deviation, N/A—non-applicable. a—low risk vs. medium risk. b—low risk vs. high risk. c—medium risk vs. high risk.

**Table 6:** The overall survival odds ratios for GRAM risk stratification.

Survival		No	Yes	OR (Univariable)	OR (Multivariable)
Group	low	77 (14.2)	464 (85.8)	-	-
	medium	105 (58.3)	75 (41.7)	0.12 (0.08–0.17, *p* < 0.001)	0.12 (0.08–0.17, *p* < 0.001)
	high	91 (80.5)	22 (19.5)	0.04 (0.02–0.07, *p* < 0.001)	0.04 (0.02–0.07, *p* < 0.001)

**Table 7:** Associations of individual GRAM score components with survival probability.

Dependent: Survival		No N (%)	Yes N (%)	OR (Univariable)	OR (Multivariable)
Xray abnormalities	No	73 (28.4)	184 (71.6)	-	-
	Yes	200 (34.7)	377 (65.3)	0.75 (0.54–1.03, *p* = 0.076)	0.93 (0.62–1.38, *p* = 0.722)
Hemoptysis	No	259 (32.1)	547 (67.9)	-	-
	Yes	14 (50.0)	14 (50.0)	0.47 (0.22–1.02, *p* = 0.052)	0.55 (0.22–1.41, *p* = 0.209)
Age	[65–75)	88 (30.3)	202 (69.7)	-	-
	<65	58 (19.1)	246 (80.9)	1.85 (1.27–2.71, *p* = 0.002)	1.08 (0.69–1.68, *p* = 0.745)
	≥75	127 (52.9)	113 (47.1)	0.39 (0.27–0.55, *p* < 0.001)	0.44 (0.29–0.68, *p* < 0.001)
Dyspnea	No	145 (34.3)	278 (65.7)	-	-
	Yes	128 (31.1)	283 (68.9)	1.15 (0.86–1.54, *p* = 0.335)	1.00 (0.69–1.44, *p* = 0.991)
Unconsciousness	No	165 (24.5)	509 (75.5)	-	-
	Yes	108 (67.5)	52 (32.5)	0.16 (0.11–0.23, *p* < 0.001)	0.18 (0.11–0.27, *p* < 0.001)
No of comorbidities	≥5	23 (65.7)	12 (34.3)	-	-
	0	26 (12.9)	175 (87.1)	12.90 (5.85–29.88, *p* < 0.001)	11.27 (4.54–29.05, *p* < 0.001)
	1	64 (27.6)	168 (72.4)	5.03 (2.41–11.02, *p* < 0.001)	5.23 (2.25–12.57, *p* < 0.001)
	2	64 (34.0)	124 (66.0)	3.71 (1.77–8.17, *p* = 0.001)	4.67 (2.00–11.29, *p* < 0.001)
	3	59 (50.4)	58 (49.6)	1.88 (0.87–4.25, *p* = 0.114)	2.35 (0.97–5.85, *p* = 0.061)
	4	37 (60.7)	24 (39.3)	1.24 (0.53–3.01, *p* = 0.622)	1.72 (0.65–4.64, *p* = 0.277)
NLR	<3.13	98 (21.0)	369 (79.0)	-	-
	≥3.13	175 (47.7)	192 (52.3)	0.29 (0.21–0.39, *p* < 0.001)	0.31 (0.22–0.44, *p* < 0.001)
LDH max	High	255 (34.0)	494 (66.0)	-	-
	Low	1 (20.0)	4 (80.0)	2.06 (0.30–40.50, *p* = 0.518)	1.12 (0.14–23.54, *p* = 0.924)
	Normal	17 (21.2)	63 (78.8)	1.91 (1.12–3.44, *p* = 0.022)	2.25 (1.17–4.52, *p* = 0.018)
Bilirubin	High	32 (51.6)	30 (48.4)	-	-
	Low	1 (33.3)	2 (66.7)	2.13 (0.19–47.31, *p* = 0.545)	4.67 (0.28–150.95, *p* = 0.317)
	Normal	240 (31.2)	529 (68.8)	2.35 (1.40–3.97, *p* = 0.001)	2.42 (1.27–4.62, *p* = 0.007)

(%) refers to the percentage of patients from a certain risk group; N—number of patients.

**Table 8:** The total in-hospital survival odds-ratio relations for GRAM risk stratification.

Dependent: Survive Hospital		No N (%)	Yes N (%)	OR (Univariable)	OR (Multivariable)
Group	Low	39 (7.2)	502 (92.8)	-	-
	medium	76 (42.2)	104 (57.8)	0.11 (0.07–0.16, *p* < 0.001)	0.11 (0.07–0.16, *p* < 0.001)
	High	81 (71.7)	32 (28.3)	0.03 (0.02–0.05, *p* < 0.001)	0.03 (0.02–0.05, *p* < 0.001)

(%) refers to the percentage of patients from a certain risk group; N—number of patients.

**Table 9:** Clinical non-fatal events and hospitalization outcomes in the GRAM risk strata.

		Low Risk (0–0.41)	Medium Risk (a) (0.41–0.78)	High Risk (b) (0.78–1.00)	
Variables, Units (N)		n (% of Risk Category)	n (% of Risk Category)	n (% of Risk Category)	*p*-Value (for Post Hoc Analysis)
All cause shock	Yes	31 (5.7)	50 (27.8)	46 (40.7)	<0.001 (a,b)
					
Bleeding	Yes	19 (3.5)	17 (9.4)	21 (18.6)	0.002 (a) <0.001 (b)
Heart failure					
decomposition	Yes	5 (0.9)	19 (10.6)	22 (19.5)	<0.001 (a,b)
Myocardial infarction	Yes	2 (0.4)	3 (1.7)	7 (6.2)	0.098 (a) <0.001 (b)
Neurological deficits	Yes	17 (3.1)	13 (7.2)	10 (8.8)	>0.001 (a,b)
Thrombosis	Yes	13 (2.4)	8 (4.4)	7 (6.2)	>0.001 (a,b)
Deterioration	Yes	34 (6.3)	8 (4.4)	6 (5.3)	>0.001 (a,b)

n—number of patients affected by a particular non-fatal event; a—refers to the statistical significance of comparison of the particular non-fatal event in the medium-risk group to the patients from the low-risk-group; b—refers to the statistical significance of comparison of the frequency of the particular non-fatal event in the high-risk group vs. patients from the low-risk group.

## Data Availability

The datasets used and/or analyzed during the current study are available from the corresponding author upon reasonable request.
